# Mutual insurance in the entrepreneurial landscape

**DOI:** 10.1186/s13731-022-00223-6

**Published:** 2022-03-12

**Authors:** Irina L. Logvinova, Yury B. Rubin, Mikhail V. Lednev, Daniel P. Mozhzhukhin

**Affiliations:** 1 Department of Insurance, Moscow University for Industry and Finance “Synergy”, Moscow, Russian Federation; 2 Department of Theory and Practice of Competition, Moscow University for Industry and Finance “Synergy”, Moscow, Russian Federation

**Keywords:** Entrepreneurship, Features and functions of entrepreneurship, Mutual insurance, Mutual insurance organization, Members of mutual insurance organization

## Abstract

In this article, the authors introduce mutual insurance as a constructive component of the modern entrepreneurial landscape aimed at the protection of the wealth-related interests of the participants of the mutual insurance company (mutual insurance society, friendly society, etc.). Analyzing mutual insurance, the authors display it from the standpoint of entrepreneurship and assume that such companies (MICs) are among the insurance market actors. The specific feature of MICs is that they form the community of their members-policyholders. As far as members of each organization of this kind are its co-owners, they carry out some critical entrepreneurial activity functions. The object of this research is represented by the insurance market of the Russian Federation, through the prism of which the degree of development of MICs was demonstrated, and the barriers to its infrastructure growth were determined.

## Introduction

In contrast to commercial insurance companies, mutual insurance ones are less popular these days among the potential policyholders (Murgai et al., 2002). In the Soviet Union, mutual insurance did not exist at all, and thus, no mentions of it can be found in reference books. In modern Russia, not only the potential policyholders but even scientists and the leading specialists of the commercial insurance companies consider mutual insurance to be an old form of insurance relations or the old specific form of mutual assistance.

Meanwhile, in the world arena, this kind of insurance is highly popular and shows a permanent growth. The latest research published by International Cooperative and Mutual Insurance Federation (ICMIF) declares that in the 10-year period since the onset of the financial crisis (2007 to 2017), premium income of the global mutual and cooperative insurance sector rose by 30%, compared to 17% growth of the global insurance industry. As a result, the global shares of mutual and cooperative insurers rose from 24.0% in 2007 to 26.7% in 2017 (Global Mutual Market Share10, 2019).

These figures indicate that the development of mutual insurance is essential for the development of the modern insurance market (Verezubova, 2015). In particular, in the post-soviet countries, mutual insurance may play an important role in accelerating economic development. The state regulation of this sector cannot be effective without the recognition of mutual insurance as a kind of entrepreneurship (Kassim, 2013).

In the big scheme of things, mutual insurance is a phenomenon in business environment that has been developing and expanding for many centuries (mutual insurance societies, friendly societies, P&I Clubs, zemstvo insurance, etc.). However, essential definitions of mutual insurance are yet to be discussed. Present-day Russian reference books define mutual insurance as an agreement between a group of individuals or legal entities on loss compensation made to each other in particular shares. Along with this, it is described as one of the organizational forms of insurance coverage implying that each insured is a member of the insurance society (Raizberg et al., 2019).

In Item 2 of Article 1 of the Russian Federation (RF) Law “About Mutual Insurance” (2007), mutual insurance is defined as insurance of wealth interests of the members of mutual insurance society on the basis of mutuality by pooling resources within mutual insurance society. According to this definition, one can conclude that mutual insurance is a special case of a more general phenomenon—insurance. Some authors characterize “insurance” as a type of activity from one side and as a type of business from the other (Yuldashev, 2009). As a rule, insurance activity is defined as a system of actions in order to create an insurance fund and to make compensation for the losses to the insured in case the events specified in the agreement arise, while insurance entities are the insurance companies, mutual insurance societies, and organizations forming funds for self-insurance (Dubravská, 2015; Yuldashev & Tzvetkova, 2011).

The relevance of mutual insurance development is confirmed by the 2019 report on the insurance market (Insurance Information Institute, 2019). Its data show that from 2017 to 2019, there has been an increase in both the number of mutual insurance funds and the number of their participants. According to the report, the propensity to develop mutual insurance in that time period was due to the emerging risks of economic downturn, which mutual insurance could successfully hedge. Though, as indicated in the 2020 report (Insurance Information Institute, 2020), over the years, this trend persisted with the only exception for the type of threat—now, the main hazard to business operations is the impact of the COVID-19 pandemic. Representatives of small and medium-sized enterprises are actively trying to insure themselves against financial losses resulting from the pandemic, and insurance companies, in turn, are striving to hedge these risks through mutual insurance.

Deloitte (2019) states that the mutual insurance industry is expected to witness an increase in mergers and acquisitions. Mid-sized companies see such deals as a way to consolidate to further grow and expand their portfolio capabilities. In addition, such transactions are believed to allow for diversification of both products and risks through a larger portfolio (Hansen, & Nybakk, 2018). Overall, the mutual insurance market benefits from such transactions, because it becomes more reliable and holistic.

Similar inferences are provided by the KPMG report (2019). KPMG specialists denote that the emergence of mutual insurance has significantly transformed the insurance market. Thanks to the support of insurance technology, more participants entered the insurance market, which boosted the development potential of the entire insurance ecosystem and sharpened market competition. At the same time, there is evidence that mutual insurance has significantly reduced the level of risk in the insurance ecosystem, which has protected participants of this market from sudden bankruptcy.

According to the report by the Canadian Institute of Actuaries (2020), during the COVID-19 pandemic, insurance claims increased drastically. This applies to both individuals and companies. Given the rise in insurance payments, the issue of mutual insurance becomes more relevant than ever because mutual insurance allows one to reduce the chance of bankruptcy and distribute the risks among all market participants.

As follows from the in-depth analysis of the literary sources, the issue of mutual insurance is mainly considered in the context of that part of the market, which is represented by insurance companies. However, as the data from analyzed reports show, in the context of the unforeseen impact of the pandemic on the economy, insurance, including mutual one, is extremely important for small, medium-sized, and large businesses, such as, for instance, air carriers and logistics companies. As far as this aspect remains unexplored and requires thorough scientific development, the aim of this study was to determine the role and place of mutual insurance companies (MICs) as an existing insurance practice in the entrepreneurial landscape of the Russian Federation. This will make it possible to assess the degree of institutional development of MIC in this market.

## Methods

To achieve the ultimate study goal, data from the quarterly reports of the Central Bank of the Russian Federation *“Information on the number of MICs”*, *“Information on premiums to MICs”*, and *“Information on payments to MICs”* for 2010–2020 were collected, compared, and graphically displayed. All the information used within the investigation was checked for relevance through separate reports characterizing modern development of mutual insurance in the world (namely, reports of International Cooperative and Mutual Insurance Federation (ICMIF), Association of Friendly Societies (UK), Association of Financial Mutuals (UK), International Association of Mutual Benefit societies (AIM), and International Group of P&I Clubs). This stage allowed analyzing the infrastructure of the MIC segment.

The investigation process also implied a thorough study of the dynamics of the number of Russian MICs and the dynamics of the members of such companies (those already with membership, joined, retired, but responsible). The cash flows of Russian MICs were reflected in the ratio of premiums and payments, as well as the relative values of premiums and payments to similar indicators of 2010. This stage made it possible to assess the financial situation of Russian MICs.

Similarly, weaknesses and strengths of the existing regulatory system in relation to MICs were identified, and the draft law on the future reform of the MIC segment in the Russian Federation was analyzed with reference to international and national regulatory legal acts. The revealed conceptual contradictions and differences of approaches were displayed in the discussion section, where new ideological and organizational concepts of conducting mutual insurance activities in the existing business landscape were demonstrated.

## Results

**Fig. 1:**
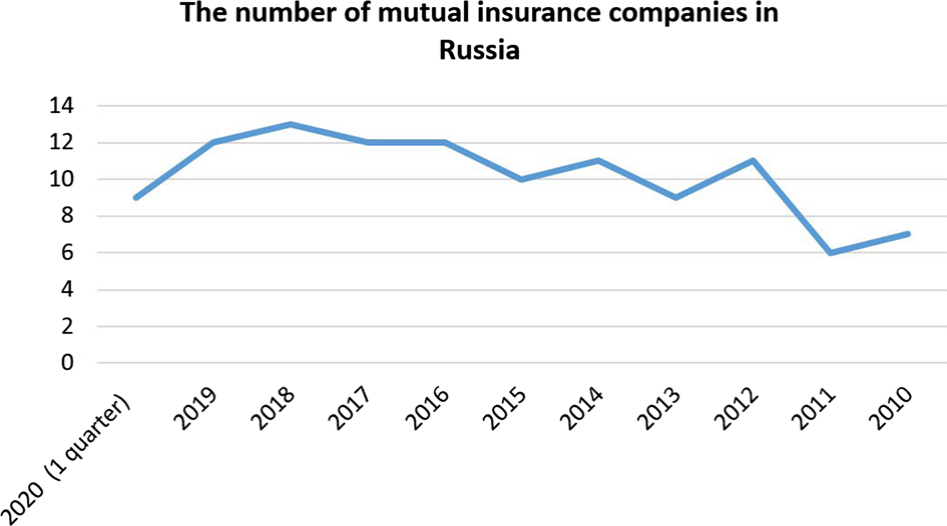
Yearly dynamics of the number of Russian MICs

As of this date, regulation, control, and supervision of the activities of insurance entities in the RF (insurance organizations, insurance brokers, and MICs) are carried out by the Bank of Russia. Although MICs are not that popular in Russia as in other world states, there is a fairly stable number of them representing the infrastructure “backbone” of the segment (Fig. 1).

**Fig. 2:**
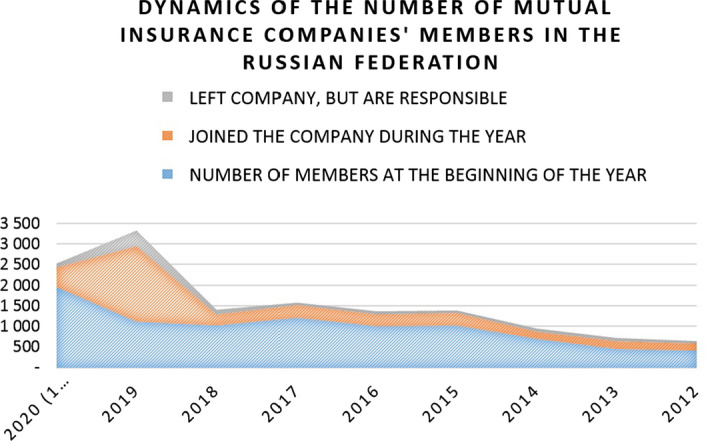
Dynamics of the number of Russian MICs’ members

In the meantime, one should not think that the segment is fully oligopolized since its structure is constantly changing—new entities attracting new members are formed in place of the liquidated ones. And the overall number of MIC participants is growing every year (Fig. 2).

**Fig. 3:**
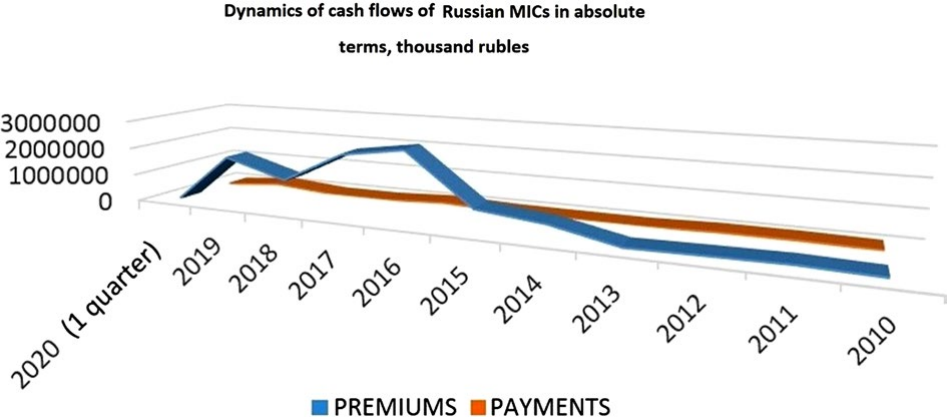
Cash flows of Russian MICs in absolute terms

Comparison of the dynamics of the number of MIC and their members makes it obvious that the trends do not coincide; that is, the liquidation of some companies has no notable effect on the general dynamics of the segment’s attractiveness for participants. This indicates, first of all, that consumers of MICs’ insurance products are a fairly stable socio-demographic category, whose preferences are weakly dependent on segment conditions. If compared with European MIC experience, a quantitative meagerness of Russian companies becomes evident. Such a market situation, where the segment demonstrates an increase in the number of consumers and their loyalty despite the minimal infrastructure development, confirms the thesis that in the modern economic realities of the Russian Federation, MICs are small but valuable elements of the country’s business landscape. An important characteristic of the segment is the quantitative dynamic structure of premiums and payments made by MICs during the year (Fig. 3).

**Fig. 4:**
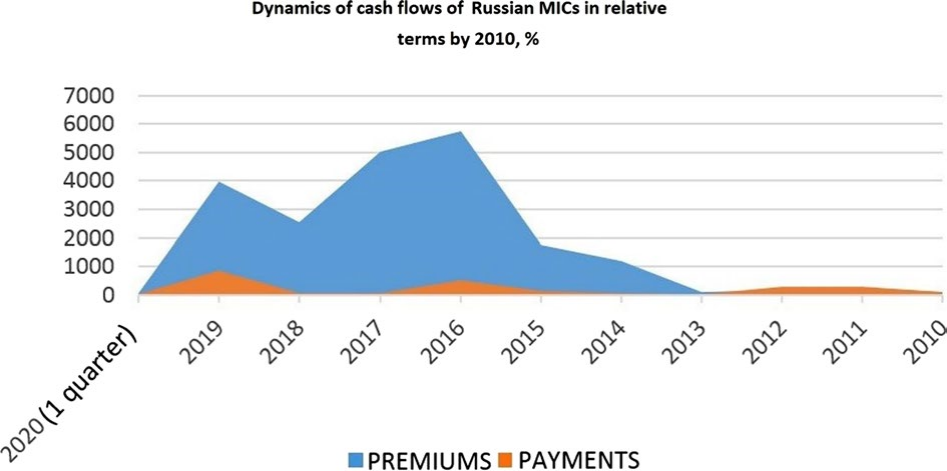
Cash flows of Russian MICs in relative terms

As it follows from the diagram above, bonuses and payments of Russian MICs were almost equal from the moment of the first assessment of the MIC segment by the Central Bank of the Russian Federation to the year 2013. The active consumption of insurance products (that is, the direct formation of premiums) began in 2014 when the Russian economy faced economic sanctions, devaluation of the ruble, and a decrease in domestic national demand. Though, the same cannot be said for the dynamics of payments. Correspondingly, the collected data presume that the weak infrastructural development of the MIC segment is associated with insufficient demand and imbalances in the demand and supply for insurance products. With respect to the above, it seems reasonable to compare premiums and payments in relative terms to the primary estimates of the segment’s structure made by the Central Bank (Fig. 4).

Figure 4 makes it clear that during the initial stages of the segment’s development, payments in relative values grew faster than premiums, which testifies to the effectiveness of the mechanism created in relation to MICs’ members. At the same time, the dynamics of relative values for premiums demonstrate a growth significantly greater than inflation and ruble devaluation indicators for the same period. In other words, the growth of the incoming cash flow in the segment is connected not with the volatility of financial markets but with the transformation of consumer behavior (including the formation of loyalty).

The quantitative data processing enabled concluding that the barriers to the institutional development of Russian MICs are not in the field of supply and demand, financial markets, consumer relations, and the comparability of premiums and payments. The authors of this paper suggest that the reasons for the weak infrastructural growth of MICs in the existing entrepreneurial landscape of the Russian Federation with the formed steady demand for insurance products lie in the plane of the regulatory policy and legal tradition of the Russian state.

The situation with mutual insurance companies is very similar to that with credit unions. In both cases, there are non-profit organizations serving their members, and there is still no federal law regulating their activities.

At present, the activities of MICs are regulated by Article 968 of the Civil Code of the Russian Federation. This article refers to the law on mutual insurance, but such a law has not yet been adopted. Another feature of the legal status of MICs is that if they are limited to insuring only their members, they do not need a license. The legal framework for the activities of MICs is rather contradictory, and there is no uniform practice in the application and interpretation of normative acts. This creates serious obstacles to the normal functioning of mutual insurance societies.

Since these companies do not aim at a profit, their services are, by definition, cheaper than the services of commercial insurance. However, public funds should be sufficient to cover risks, which is often difficult at the initial stage of their activity. The principle of reciprocity has undeniable advantages for solving the issue of reliability. In most cases, MIC’s members are personally known to each other, which provides a high level of trust in combination with real mechanisms for monitoring the use of insurance reserves. Trusting relationships enable greater flexibility in various aspects of the activities of the MIC. Hence, for example, a MIC can use part of its insurance reserves to implement development programs for its members (like lending or investment projects). An additional plus is an absence in the legislation of requirements for the size of the minimum authorized capital and the fact that the legislation does not establish the size of the minimum entry fee to the MIC. This makes it possible to create new mutual insurance companies relatively quickly and cheaply.

In addition to the possible lack of funds at the initial stage of work, the problem area may also be represented by the sufficient qualification of the members of a MIC in the insurance business. In truth, a MIC needs to hire professionals or use the services of a special company, which can either be created by the company or invited from the outside on a commercial basis. In the literature, such companies are called managers; however, unlike management companies of mutual investment funds, they manage not financial assets with the goal of their growth but risks through redistributive insurance operations.

In 2019, the Ministry of Finance of the Russian Federation prepared a draft amendment to the law on MICs, which was designed to expand the number of operating organizations of this class of insurance market entities in the Russian Federation. As for now, there are only 9 MICs in Russia, while in a number of other countries, such organizations not only compete with insurers but also surpass them in financial power. The implementation of the amendments will provide new opportunities for policyholders, and first of all for associations created for professional interests, whose members are instructed by lawmakers to ensure liability for damage to third parties in the performance of their official duties.

The little change in the number of MICs in the Russian Federation for many years may be provoked by the fact that the requirements for MICs are comparable to the requirements for insurers, and they are difficult to fulfill. Since the main vector of interests of MIC’s members is in a different field of activity, the draft amendment proposes simplifying demands to reporting and MICs’ organization. Even though a license from Central Bank to conduct MIC operations will continue to be a must, it can be obtained, for example, by the self-regulatory organization (SRO) of actuaries or another non-profit association. In order to prepare reports or perform other special tasks, a MIC may eventually receive the right to engage assistants on an outsourcing basis. In such a manner, market participants may unite and set up their own SROs in case of failure to find adequate proposals. Thus, MICs will provide new opportunities for organizing insurance coverage for small and medium-sized businesses.

The provisions of the law on consumer cooperation also apply to MICs. Accordingly, ensuring a stable financial position of the company is to be achieved through the subsidiary liability of its members. In the event of a negative financial result of mutual insurance, it becomes necessary to make an additional contribution by the MIC’s members. Implementation of the provisions of the draft will allow MICs to attract new participants, develop new types of insurance, and, as a result, satisfy the insurance needs of their members in full and at an optimal price. Although a MIC is usually created in the form of a non-profit organization, the legislation of the Russian Federation also allows it to be formed as a result of the reorganization of existing companies. Apart from this, the draft specifies conditions for voluntary withdrawal from the society, according to which cases of deliberate termination of membership in the society for the purpose of evasion from obligations are excluded. MICs work under the principles that are simplistically compared with the principles of operation of the mutual assistance cash desk. Participants’ insurance premiums do not burn out at the end of the insurance period; members of the MIC calculate the required premium amount by themselves. If this amount is not enough to cover the loss, they collect additional fees.

According to the draft, MIC can be created at the initiative of at least five individuals or at least three legal entities. For companies, members of which are both individuals and legal entities, a minimum number of participants is established as no less than five in total. In order to increase the availability of mutual insurance, the draft removes the current limitation of the Law *“On Mutual Insurance”* for the maximum allowable number of members of the company (no more than 2 thousand people). The draft establishes the requirement for separate accounting for insurance reserves of a MIC and specifies the types of contributions of company members. In particular, it eliminates the obligation for MIC members to pay a share fee and a contribution to cover costs associated with the statutory activities of the company.

In particular, the obligation to pay members of the company a share contribution and a contribution to cover expenses related to the charter activities of the company is excluded.

The draft law defines the principles of managing an MIC, the conditions for holding regular and extraordinary meetings, the procedure for preparing statutes and amending them, the requirements for preparing reports for the Central Bank, and the right to use a recourse claim against the guilty party for the loss. It also clarifies the features of information disclosure, responsibility for the misuse of personal data, the right to unite MICs to participate in the discussion of regulatory innovations, and other principles of MICs’ activities. According to the conclusion of the Ministry of Finance of the Russian Federation, the implementation of this draft will not require additional funding from the federal budget and other budgets of the Russian Federation.

## Discussion

In the insurance markets of most of the countries of the world, only two types of insurance companies can be noted: mutual (MICs) and commercial ones (CICs) (Logvinova, 2010). Here, it is important to distinguish the notions “a type of insurance company” and “a registered form of the insurance company”. While belonging to one company type, the insurers can have different registered forms as business entities (Vobly, 1993). Official MICs appeared about three thousand years ago (Kolomin & Shakhov, 1991). Communities began to apply mutual insurance to their members and officially register insurance economic entities of a certain form from about the end of the XVII century. But now, one can see different MIC forms. Some of them exist in many countries (mutual insurance societies, insurance cooperatives), although they may have certain features depending on the legislation (Association of Friendly Societies, 2020; P&I Clubs International Group, 2020), and others exist only in a particular country, such as friendly societies in Great Britain (Association of Financial Mutuals, 2020).

It is obvious that commercial insurance is an entrepreneurial activity carried on by commercial insurance organizations. When an insurance product is created by the CIC, the policyholder is only a buyer (Danish et al., 2019). An insurance agreement gives him/her neither the right nor opportunity to take part in the process of management of the payment. This right belongs to a commercial insurance entity presented by its owners. The full responsibility for obligations on insurance payments carries the insurer—the only one to manage the company’s resources. Thus, in this case, the entrepreneurial activity is carried on by the insurer (the owners of a CIC). The policyholders do not take part in it.

In the frames of mutual insurance, the situation is different. The creation of an MIC is always initiated by the potential policyholders (persons or legal entities) to create insurance products for themselves (Odierno, 2013). They join their resources and formalize their relations in an agreement specifying rules of MIC’s creation and insurance payment. Hence, the insurance fund is formed at the expense of the resources of each MIC member as joint property. Policyholders do not have a right of sole management and control of the fund but can participate in management and control over the fund as well as to use its resources. Similarly, each policyholder is responsible for obligations related to the creation of insurance products using the resources of this fund, and this liability is distributed between the insurer (MIC) and the policyholders (MIC members).

The right of each policyholder to be the co-owner of MIC’s resources is inextricably linked with the joint responsibility related to insurance obligations. The insurer—MIC—has the responsibility for obligations related to the materialization of an insurance product. However, if the amount of resources in the insurance fund is not sufficient to fulfill the insurance obligations, all the policyholders—members of this organization—have joint subsidiary responsibility for this obligation (Association of Friendly Societies, 2020; International Group of P&I Clubs, 2020). This obligation is materialized in the decision of all the members to make an additional payment to the organization, which must be approved by the majority of members. Provided that MIC members participate in resource creation and management and a MIC creates insurance products, all the parties involved become entrepreneurs. Furthermore, given that all MIC insurers are its co-owners and service users concurrently, the interests of such persons unite the interests of both the insured and the rulers. On the one hand, they are focused on the provision of quality insurance protection of their wealth interests and, on the other, on a decrease of costs of doing business (in reasonable limits).

As a rule, MICs are considered to be non-profit organizations. According to this definition, some authors conclude that organizations of this kind do not have profit and cannot have it (Safuanov et al., 2009). In our opinion, MIC, like any other business entity, cannot develop without gaining profit. It is considered a non-profit entity because profit is not the main aim of its activity and is not distributed between its co-owners. For the MIC, profit is, possibly, the most important condition for achieving the company’s primary goal—insurance protection of property interests of its participants on terms that they consider more attractive than those of a commercial insurer. And it is reasonable that the profit is needed to pay off the trust.

By and large, MIC’s profit is spent on the achievement of the general aim, for the sake of which individuals and legal entities unite. It should be more correct to call it the benefit that MIC members get from receiving insurance products in more advantageous conditions (Lupova-Henry et al., 2021). The advantage is, for example, an opportunity to reduce the size of insurance premiums or even get insurance products free of charge in some cases. If the financial recourses of the community exceed its obligations for a certain period of time, MIC members (or their representatives) can decide to decrease the sum of insurance payments for some members or even abolish it fully (Logvinova, 2014). In the case of buying the same insurance products in a CIC, this scenario is absolutely impossible.

MIC members jointly carry subsidiary responsibility for the obligations of the organization. Subsequently, there is always a possibility that they have to make additional payments for discharge of MIC obligations or will get less reimbursement than anticipated. At the same time, MIC members have the ability to manage such risks (Tikhomirov et al., 2017). They can decide to take preventive measures to reduce the likelihood of an insured event occurring or to reduce the possible damage from such an event. The main thing is that the community of insureds, the MIC participants, usually make a wise decision to allocate a certain amount of money for this purpose. Thus, insurers as co-owners of MICs express an interest in minimizing the cost of doing business (Rubin et al., 2018).

As the actors of the insurance market, MICs act in a competitive environment (Ujunwa & Modebe, 2011). At the beginning of the XX century, there were two types of competition in the insurance market. The first is competition between insurance companies of the same kind (between CICs), whereas the second is competition between insurance companies of different profiles (between CICs and MICs). However, today we can add another category to this list, implying competition between MICs (Logvinova, 2010).

In sum, competition can be defined as a system of professional engagement and interaction of subjects of any activity type (economic, scientific, sports, creative, domestic, etc.) with rivals. It consists of specific actions of each of the rivals in relation to each other (Rubin et al., 2017). According to this approach, CICs and MICs can be not only rivals but also partners. Sometimes they oppose each other, but they can also cooperate on a mutually beneficial basis. For example, MICs tend to reinsure their risks with CICs.

## Conclusion

The practice of MICs in Russia has officially existed for 10 years, but with the actual growth of insurance service users and companies’ participants, the number of companies remains virtually unchanged. The reason for this, first of all, lies in the regulatory policy and the specifics of the organizational activities of MICs in the Russian Federation, which are expected to be tackled in the near future with the adoption of the corresponding legal reform. In general, this study showed that the current state of MIC in Russia is relatively stable: the number of MICs grew from 7 in 2010 to 9–13 in 2018–2020, the number of MIC participants grew from 500 in 2012 to 2500 in 2020, and the money turnover grew up to 4000% in 2019 compared with 2010. It was revealed that MICs occupy an important place in the entrepreneurial landscape of the Russian Federation. However, despite the recent activation of this type of insurance, one should not overestimate its growth rates and expect that MICs in Russia will be able to compete with CICs in the foreseeable future. Since MIC are aimed at small and partly medium-sized insurers who, for one reason or another, do not have the services of insurance companies, they are unable to raise as many funds as are accumulated by commercial insurance companies. The main MIC activity barriers reside in the plane of the activity format.

However, there are sectors of the insurance market that are, by definition, intended for MICs. It is primarily about the insurance of homogeneous risks. Examples include professional liability insurance of notaries, doctors, lawyers, individual entrepreneurs. Their insurance through MICs has good prospects, which is proved by the world reports (Deloitte, 2019; KPMG, 2019). These groups of entrepreneurs need inexpensive and reliable insurance, and insurance interests are similar. It is important that they receive a sufficiently high and stable income that will allow them to create viable MIC if they wish. Today, there are ideological contradictions in the “road map” of the MIC activity, where only coordination of norm-setting with market participants and entrepreneurs will allow creating an effective interaction mechanism.

The mutual insurance sector in Russia occupies an important place in business activity, as it allows one to hedge many emerging risks (which is proved by the grooving number of MIC participants). At the same time, the analyzed sources confirm the flourishing perspectives of mutual insurance, which is in line with the present study results. The purpose of mutual insurance is to minimize threats, which is one of the key aspects of long-term company management. The authors assume that in the near future, the tendency to develop and increase the volume of the mutual insurance market in the Russian Federation will continue.

### Research limitations

The research results are limited by the fact that the methodology of collecting and processing quantitative data by the Central Bank could change from 2010 to 2020, thereby distorting the correct reflection of the economic reality of the Russian Federation. In addition, until 2010, data on MIC as a segment were not collected, although, in fact, there was such an activity. For the period 2010–2011, there were no available data on the number of MIC members, their entry into the company, and withdrawal from it.

Because of these limitations, important questions have arisen concerning the peculiarities of mutual insurance as entrepreneurial activity in various insurance cases (property insurance, liability insurance, etc.). Therefore, further research and academic discussions are needed on peculiarities of such activity in realization of interests of insurants and insurers, special approaches to state regulation of such activity, application of mutual insurance in the field of obligatory insurance.

## Data Availability

Data will be available on request.
